# Changing Admission Patterns in Pediatric Emergency Departments during the COVID-19 Pandemic in Italy Were Due to Reductions in Inappropriate Accesses

**DOI:** 10.3390/children8110962

**Published:** 2021-10-25

**Authors:** Ivana Rabbone, Francesco Tagliaferri, Elena Carboni, Beatrice Crotti, Jessica Ruggiero, Alice Monzani, Lorenza Bonetti, Martina Soliani, Simonetta Bellone, Claudio Cavalli, Andrea E. Scaramuzza

**Affiliations:** 1 Division of Pediatrics, Department of Health Sciences, University of Piemonte Orientale, 28100 Novara, Italy; ivana.rabbone@uniupo.it (I.R.); cecco.taglia@gmail.com (F.T.); alice.monzani@gmail.com (A.M.); simonetta.bellone@med.uniupo.it (S.B.); 2 Pediatric Unit, ASST Cremona, 26100 Cremona, Italy; elena.carboni@yahoo.it (E.C.); crobea@hotmail.it (B.C.); jeca.jr92@gmail.com (J.R.); l.bonetti@asst-cremona.it (L.B.); martina.soliani@asst-cremona.it (M.S.); claudio.cavalli@asst-cremona.it (C.C.)

**Keywords:** COVID-19, pediatrics, emergency department, inappropriate accesses

## Abstract

During the initial phase of the national lockdown, we found that there were sharp decreases in admissions to two pediatric emergency departments (EDs) in northern Italy (Cremona and Novara). Here we present a detailed analysis of these admission patterns and types of admissions over a longer timeframe. ED admissions data were anonymously extracted from the departmental management software. Admissions data from 2019 and 2020 were analyzed and compared separately for each ED and combined. There was a 73.2% decrease in total admissions compared with the same period in 2019. With respect to admission diagnoses, there was a significant (*p* < 0.001) drop in infectious (−51%), respiratory (−25.5%), and nervous systems diseases (−50%) and injuries and poisoning (−17%) but not endocrine, metabolic, neoplastic, circulatory, or musculoskeletal diseases. White codes (patients with minor injuries for whom ED medical care is not required) significantly decreased by 56.3% (*p* < 0.001). Even if the COVID-19 pandemic represented an enormous healthcare burden in Italy, especially during the first months of the pandemic (late February to May), the workload of pediatric EDs was significantly reduced, especially for unnecessary accesses (white codes).

## 1. Introduction

COVID-19 (coronavirus disease 2019) probably started in Wuhan, China at the end of 2019, spreading globally over the following months. Despite the roll-out of vaccination programs and improved therapy, the disease continues to incur significant healthcare and societal burdens worldwide [1]. After so-called “Patient 1” was admitted to the intensive care unit of his local hospital due to a deteriorating clinical condition from SARS-CoV-2/COVID-19 infection, Italy has been among the most severely affected countries worldwide both in terms of numbers of infected people and deaths.

On 9 March 2020 (effective starting the following day), the whole of Italy received the “#stayhome” (#iostoacasa) executive order from the Italian Government [2]. This order was set to remain in place until after Easter, with all public gatherings banned and travel only allowed for “urgent, verifiable work situations and emergencies or health reasons”. However, due to the continuing severe situation, the order deadline was postponed to 4 May 2020. After that, work activities slowly returned to normal, albeit with some restrictions until 3 June 2020, when all activities returned normal including movement within regions and, on 14 June 2020, between regions.

A common finding during the pandemic was a dramatic change in admission rates (73–88% reduction) to pediatric emergency departments (EDs), as noted by Lazzerini et al. [3] and others [4,5,6], including ourselves [7]. In addition to an aversion to attending EDs due to a fear of contracting SARS-CoV-2, a lack of preparedness (e.g., short supplies of personal protective equipment for healthcare workers, hospital equipment, and sanitizing supplies) contributed to the struggles experienced by healthcare facilities around the world [8] and could have had an impact on the number of patients with chronic diseases attending EDs. However, these studies were conducted in the first weeks of the pandemic and usually lacked details about the reasons children presented to the EDs.

Recognizing this, here we present data on admission patterns during the lockdown and immediately after to two pediatric EDs in Northern Italy: Cremona (72,680 inhabitants), which was the center of the COVID-19 epidemic in Italy, and Novara (104,268 inhabitants), close to Lombardy. The Novara and Cremona pediatric EDs are full-service 15 bed facilities that provide emergency services for children 0 to 14 years of age. These pediatric EDs typically serve about 15,000 and 9000 patients per year, respectively. The aim of the study was to report pediatric ED utilization during the COVID-19 pandemic at these two pediatric referral centers located close to the epicenter of the pandemic in northern Italy, evaluating both the level of urgency of service utilization and the presenting diagnoses.

## 2. Materials and Methods

We retrospectively collected pediatric ED admission data from 20 February to 2 June 2020 and compared these data with the same data registered over the same period in 2019. ED admission data were anonymously extracted from ED management software. The day and time of admission, admission color code, time of discharge, outcome, and the diagnosis were collected. The color coding used at triage was: red code (emergency, intervention time 0 min), patients cannot survive without immediate treatment but have a chance of survival; yellow code (urgency, 15 min), patients have at least one vital function compromised, but their condition is currently stable and they are not in immediate danger of death; green code (wait, 60 min), patients will need medical care at some point after more critical injuries have been treated; white code (dismiss, no time specified), patients with minor injuries for whom ED medical care is not required and should have possibly gone to their family pediatrician. The diagnoses were categorized according to the main diagnostic groups of the International Classification of Diseases, version 9 (ICD-9). Data were analyzed separately for each ED and combined, with data compared between 2019 and 2020. Comparisons were performed using the chi-squared or Fisher’s exact tests, as appropriate, using IBM SPSS Statistics v23 (Armonk, NY, USA).

Our ethics committee (document n. CE 81/20, protocol 533/CE) approved the study on 11 May 2020, and the study was conducted in line with the Declaration of Helsinki as revised in 2013.

## 3. Results

**Figure 1:**
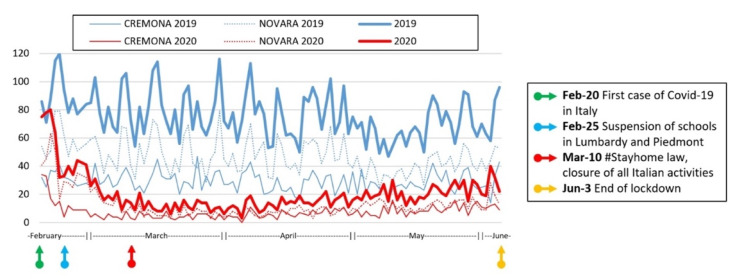
Admissions to pediatric EDs during the COVID-19 pandemic between 20 February 2020 and 2 June 2020 compared with the same period in 2019.

Overall, there was a 73.2% decrease in pediatric ED admissions between 20 February, and 3 June 2019 and the same time period in 2020, with 2112 patients admitted in 2020 compared with 7871 patients in 2019 (Figure 1). Similar trends were observed at both EDs, with a significant reduction in all triage color codes apart from red (Table 1). When examined according to age groups (less than one year of age, 1–6 years, and 7–14 years), white code admissions reduced in 1–6-year-olds and 7–14-year-olds (−24.1% and −57.3%, respectively) but not in patients less than one year of age (3% increase). Green codes were stable in younger children (−0.2%) and increased in older children (10.8% and 71.5%, respectively), while yellow codes decreased in children less than one year of age and 1–6-year-olds (−32.7% and −6.4%, respectively) but not in older children (54.9%). No difference was observed in red codes according to age group.

Remarkably, with respect to the admission diagnoses (Table 2), there was a significant reduction in admissions for infectious diseases (−50.9%, *p* < 0.001), respiratory diseases (−25.1%, *p* < 0.001), and nervous systems diseases (mainly headache; −49.7%, *p* < 0.001). Injuries and poisoning significantly increased (17%, *p* < 0.001). No difference was observed for endocrine, metabolic, neoplastic, circulatory, and musculoskeletal diseases, notwithstanding the low numbers in these categories.

Moreover, during the observation period, in the Cremona pediatric ED, 9 out of 168 nasopharyngeal swabs performed (5.4%) were COVID-19 positive. Four of these cases were admitted to the hospital for mild or moderate symptoms, representing 2.4% of children tested. In Novara, 9 out of 91 children were positive (9.9%) for COVID-19, and only 2 were admitted to the hospital.

## 4. Discussion

Here we confirmed a significant change and overall decrease in admissions to the Novara and Cremona EDs, mainly due to reductions in white code admissions. With respect to the diagnosis, there was a significant decrease in infectious (−51%), respiratory (−25.5%), and nervous system diseases (−50%) and injuries and poisoning (−17%) but not endocrine, metabolic, neoplastic, circulatory, and musculoskeletal diseases.

Observations of a drastic decrease in pediatric ED admissions during the pandemic [1,2,3,4,5] raise the question of why these decreases occurred. While possible reasons include fear of becoming infected with SARS-CoV2 in the ED or a reduction in other seasonal infections due to self-isolation, robustly answering this question is of paramount importance. 

In our previous study [7] covering a much shorter period, we observed a significant decrease in white codes (13% in 2020 vs. 30% in 2019, *p* < 0.001) at both EDs and a significant increase in green codes (84% in 2020 vs. 68% in 2019, *p* < 0.001), suggesting that most of the non-significant pathologies usually seen at our EDs were avoided. These differences in admission codes were reflected in the different age groups, with codes decreasing especially in 1–6-year-olds and 7–14-year-olds.

Many authors [3,4,5,6,7] have concluded that this observed decrease in admissions has been due to a fear of catching SARS-CoV-2 in EDs and fewer intercurrent illnesses due to school closures and reductions in personal contacts. However, our finding of white codes particularly decreasing supports a hypothesis that EDs are often used by parents for non-urgent matters as a substitute for visiting family doctors. This might be particularly true in the winter season, when seasonal infectious disease such respiratory diseases are common in children and fill outpatient clinics to capacity.

Parents may also have avoided attending hospital due to the expectation of overcrowding and long wait times, as reported for adult EDs in the Lombardy and Piedmont areas heavily affected by COVID-19 during this period [9,10,11,12].

The analysis of the ICD-9 diagnosis seems to further support this hypothesis, because similar admission rates of serious diseases (neoplastic, circulatory, endocrine, etc.) [13,14] and a significant reduction in respiratory or seasonal diseases were observed.

The Diabetes Study Group of the Italian Society for Pediatric Endocrinology and Diabetology demonstrated that, during the lockdown, there were 23% fewer new diabetes cases compared to the same period in 2019 and that children presenting with DKA had more severe DKA (pH < 7.1) in 2020 than in 2019 (*p* = 0.03), although no diagnoses were missed overall [15]. Similar findings have been reported by other authors for other serious diseases [16].

Similar findings have been observed worldwide both in adult and pediatric patients. Rennert-May et al. [17] reported a significant reduction in daily medical and surgical admissions of adult patients through the ED in Alberta, Canada, after the implementation of COVID-19 public health measures. Comparable conclusions have been described in Finland [18], Thailand [19], and Croatia [20]. 

In an interesting multicenter analysis and review of the literature about the impact of the lockdown on pediatric EDs in the Netherlands and worldwide, EDs accesses in the Netherlands reduced by 59% and worldwide by 30–89% [21]. Moreover, care utilization for noninfectious diagnoses decreased, which may indicate that there has been significant pediatric care avoidance during the pandemic [21]. 

Our data suggest not only that the impact of the COVID-19 pandemic was to dramatically limit inappropriate accesses (white codes) but also that it may have resulted in some serious illnesses remaining undiagnosed. In this respect, it might be beneficial to educate parents about when go to EDs and the use of telemedicine, where possible, as shown for patients with type 1 diabetes [22].

ED overcrowding is a global problem that has a massive organizational and fiscal impacts, hampering the ability to provide critical services to patients suffering from medical emergencies in a timely manner in at- or over-capacity departments. After the COVID-19 pandemic has resolved, the best way to disincentivize inappropriate use of EDs will require serious consideration. Possible solutions might include education about medical necessity for inappropriate users, expansion of the pre-hospital role of primary care and inappropriate use prevention, and improved access to alternative healthcare services. Health education programs about the most common childhood diseases could also be of help, maybe together with specifically prepared leaflets or electronic forms. 

One disincentive might be the requirement to a fee to pay if pediatric ED access is recognized as inappropriate (i.e., a white code). This already occurs in Italy for older children (aged 14 and over) and adults but, given our findings, might also be appropriate for all pediatric patients. If coupled with education, this could result in more conscious resource allocation or utilization. The payment system based upon diagnosis-related groups (DRGs) using the third payer model introduced in Italy about 15 years ago has made optimizing the use of hospitals and EDs a target of increasing concern [23]. 

The lower cost of inappropriate admissions might suggest that they are less of a problem in economic terms. However, focusing on costs alone does not consider the perverse effects on the rational use of resources, considering that these patients are often examined as a priority by the attending physician to exclude a severe condition and to discharge the patient as quickly as possible. Furthermore, every admission is associated with risks, and it is highly improbable that the experience of attending the ED would improve quality of life for the patient [24,25]. 

Our study has some limitations. The retrospective nature of the study did not allow us to look more detail at the different types of accesses, with only an aggregated analysis of data possible. There were only a few admissions for some diagnostic categories, so the differences observed for these categories must be interpreted with caution. The analysis covers the period of the most stringent lockdown in Italy, so may not reflect subsequent periods with less closures and restrictions. Finally, the comparison of two time points using aggregate data might result in bias.

## 5. Conclusions

In conclusion, even if in Italy the COVID-19 pandemic represented an enormous burden, especially during the few first months (late February until May 2020), the workload of pediatric EDs significantly reduced, especially for unnecessary accesses (white color codes). Admissions for serious diseases were unaffected by COVID-19, even if a delay in ED access was observed in some instances. Our results may have important implications for public health experts and policymakers to re-shape the emergency medical care system to avoid unnecessary ED attendances.

## Figures and Tables

**Table 1:** Admissions data for children under 15 years of age attending the Cremona and Novara pediatric EDs during the COVID-19 pandemic between 20 February 2020 and 2 June 2020 compared with the same period in 2019.

	NOVARA				CREMONA				MERGED DATA			
	2019	2020	% Change	*p*-Value	2019	2020	% Change	*p*-Value	2019	2020	% Change	*p*-Value
**Admissions (n)**	4869	1337	−72.5%		3002	775	−74.2%		7871	2112	−73.2%	
***Triage***												
White (n)	859	114	−86.7%		1514	164	−89.2%		2373	278	−88.3%	
White (%)	17.64%	8.53%	−51.7%	**<0.001**	50.43%	21.16%	−58.0%	**<0.001**	30.15%	13.16%	−56.3%	**<0.001**
Green (n)	3900	1181	−69.7%		1466	599	−59.1%		5366	1780	−66.8%	
Green (%)	80.10%	88.33%	10.3%	**<0.001**	48.83%	77.29%	58.3%	**<0.001**	68.17%	84.28%	23.6%	**<0.001**
Yellow (n)	101	39	−61.4%		22	12	−45.5%		123	51	−58.5%	
Yellow (%)	2.07%	2.92%	40.6%	n.s.	0.73%	1.55%	111.3%	**<0.05**	1.56%	2.41%	54.5%	**<0.01**
Red (n)	2	1	−50.0%		0	0	-		2	1	−50.0%	
Red (%)	0.04%	0.07%	82.1%	n.s.	0.00%	0.00%	-	n.a.	0.03%	0.05%	86.3%	n.s
**Outcome**												
Discharge to home (n)	4615	1206	−73.9%		2710	663	−75.5%		7325	1869	−74.5%	
Discharge to home (%)	94.78%	90.20%	−4.8%	**<0.001**	90.27%	85.55%	−5.2%	**<0.001**	93.06%	88.49%	−4.9%	**<0.001**
Hospital admission (n)	110	101	−8.2%		230	107	−53.5%		340	208	−38.8%	
Hospital admission (%)	2.26%	7.55%	234.4%	**<0.001**	7.66%	13.81%	80.2%	**<0.001**	4.32%	9.85%	128.0%	**<0.001**
Other * (n)	144	30	−79.2%		62	5	−91.9%		206	35	−83.0%	
Other (%)	2.96%	2.24%	−24.1%	n.s.	2.07%	0.65%	−68.8%	**<0.01**	2.62%	1.66%	−36.7%	**<0.05**

* Other means discharged home without further follow-up, transferred to another hospital due to a lack of beds, or because the patient needed care that was not available in the hospital.

**Table 2:** Diagnoses for children under 15 years of age attending the Cremona and Novara pediatric EDs during the COVID-19 pandemic between 20 February 2020 and 2 June 2020 compared with the same period in 2019. Diagnoses are grouped according to the main ICD-9 diagnostic categories. Note: percentage changes are calculated from the percentages in each year rather than the absolute numbers.

	NOVARA				CREMONA				MERGED DATA			
Diagnostic Categories (ICD-9)	2019 (%)	2020 (%)	% Change	*p*-Value	2019 (%)	2020 (%)	% Change	*p*-Value	2019 (%)	2020 (%)	% Change	*p*-Value
1. Infectious and parasitic diseases (001–139)	513 (10.5)	65 (4.9)	53.3%	**<0.001**	11 (0.37)	4 (0.52)	40.5%	n.s.	524 (6.66)	69 (3.27)	−50.9%	**<0.001**
2. Neoplasms (140–239)	2 (0.04)	0 (0)	−100%	n.s.	0 (0)	1 (0.13)	0.13%	**<0.05**	2 (0.02)	1 (0.05)	136%	n.s.
3. Endocrine, nutritional and metabolic diseases, and immunity disorders (240–279)	7 (0.14)	9 (0.67)	28.6%	n.s.	8 (0.27)	1 (0.13)	−51.8%	**n.s.**	15 (0.19)	10 (0.47)	28.3%	**<0.05**
4. Diseases of the blood and blood-forming organs (280–289)	7 (0.14)	3 (0.22)	57.1%	n.s.	8 (0.27)	2 (0.26)	−3%	n.s.	15 (0.19)	5 (0.24)	24.6%	n.s.
5. Mental disorders (290–319)	5 (0.11)	5 (0.38)	245%	**<0.05**	26 (0.87)	9 (1.16)	33.5%	n.s.	31 (0.39)	14 (0.66)	70%	n.s.
6. Diseases of the nervous system and sense organs (320–389)	277 (5.69)	42 (3.14)	−44.8%	**<0.001**	249 (8.29)	29 (3.74)	−54.9%	**<0.001**	526 (6.69)	71 (3.36)	−49.7%	**<0.001**
7. Diseases of the circulatory system (390–459)	6 (0.12)	3 (0.22)	87%	n.s.	7 (0.23)	0 (0)	−100%	n.s.	13 (0.16)	3 (0.14)	−11.2%	n.s.
8. Diseases of the respiratory system (460–519)	1160 (23.8)	264 (19.7)	−17%	**<0.01**	574 (19.1)	84 (10.8)	−4.23%	**<0.001**	1734 (22)	348 (16.5)	−25.1%	**<0.001**
9. Diseases of the digestive system (520–579)	163 (3.35)	52 (3.89)	16.1%	n.s.	143 (4.76)	38 (4.9)	2.9%	n.s.	306 (3.89)	90 (4.26)	9.5%	n.s.
10. Diseases of the genitourinary system (580–629)	68 (1.4)	36 (2.69)	92.3%	**<0.01**	72 (2.4)	16 (2.1)	−14%	n.s.	140 (1.78)	52 (2.46)	38.3%	**<0.05**
11. Complications of pregnancy, childbirth, and the puerperium (630–679)	1 (0.02)	1 (0.07)	273%	n.s.	0 (0)	2 (0.26)	100%	**<0.01**	1 (0.01)	3 (0.14)	132%	**<0.01**
12. Diseases of the skin and subcutaneous tissue (680–709)	122 (2.5)	32 (2.39)	−4.3%	n.s.	140 (4.66)	29 (3.74)	−19,7%	n.s.	262 (3.33)	61 (2.89)	−13.3%	n.s.
13. Diseases of the musculoskeletal system and connective tissue (710–739)	56 (1.15)	14 (1.05)	−8.9%	n.s.	61 (2.03)	12 (1.55)	−23.7%	n.s.	117 (1.49)	26 (1.23)	−17.4%	n.s.
14. Congenital anomalies (740–759)	18 (0.34)	8 (0.61)	61%	n.s.	35 (1.19)	16 (2.11)	77%	n.s.	53 (0.69)	24 (1.16)	68%	**<0.05**
15. Certain conditions originating in the perinatal period (760–779)	8 (0.17)	2 (0.15)	−10%	n.s.	4 (0.14)	3 (0.4)	191%	n.s.	12 (0.16)	5 (0.24)	55%	n.s.
16. Symptoms, signs, and ill-defined conditions (780–799)	1126 (23.83)	360 (27.54)	16%	**<0.01**	572 (19.44)	194 (25.59)	32%	**<0.001**	1698 (22.15)	554 (26.83)	21%	**<0.001**
17. Injury and poisoning (800–999)	1150 (24.34)	397 (30.37)	25%	**<0.001**	951 (32.32)	267 (35.22)	9%	n.s.	2101 (27.4)	664 (32.15)	17%	**<0.001**
Supplementary classification of factors influencing health status and contact with health services (v01–v89)	30 (0.63)	10 (0.77)	20%	n.s.	4 (0.14)	6 (0.79)	482%	**<0.01**	34 (0.44)	16 (0.77)	75%	n.s.
Supplementary classification of external causes of injury and poisoning (e800–e999)	6 (0.13)	4 (0.31)	141%	n.s.	77 (2.62)	45 (5.94)	127%	**<0.001**	83 (1.08)	49 (2.37)	119%	**<0.001**
Total admissions with diagnosis	4725	1307			2942	758			7667	2065		

## Data Availability

Data are available upon request.
